# A Novel Two-Stage Illumination Estimation Framework for Expression Recognition

**DOI:** 10.1155/2014/565389

**Published:** 2014-04-13

**Authors:** Zheng Zhang, Guozhi Song, Jigang Wu

**Affiliations:** School of Computer Science and Software Engineering, Tianjin Polytechnic University, Tianjin 300387, China

## Abstract

One of the critical issues for facial expression recognition is to eliminate the negative effect caused by variant poses and illuminations. In this paper a two-stage illumination estimation framework is proposed based on three-dimensional representative face and clustering, which can estimate illumination directions under a series of poses. First, 256 training 3D face models are adaptively categorized into a certain amount of facial structure types by *k*-means clustering to group people with similar facial appearance into clusters. Then the representative face of each cluster is generated to represent the facial appearance type of that cluster. Our training set is obtained by rotating all representative faces to a certain pose, illuminating them with a series of different illumination conditions, and then projecting them into two-dimensional images. Finally the saltire-over-cross feature is selected to train a group of SVM classifiers and satisfactory performance is achieved when estimating a number of test sets including images generated from 64 3D face models kept for testing, CAS-PEAL face database, CMU PIE database, and a small test set created by ourselves. Compared with other related works, our method is subject independent and has less computational complexity *O*(*C* × *N*) without 3D facial reconstruction.

## 1. Introduction

In the last few years, with the rapid progress of human-computer intelligent interaction (HCII), automatic facial expression recognition has become a very active topic in machine vision community. Although recognition on frontal face with indoor lighting is already relatively mature, the performance among different PI is still far from satisfactory [1, 2]. So it is currently a key issue to eliminate the negative effect caused by variant PI in expression recognition.

In order to eliminate the negative effects of variant PI in expression recognition, we have to estimate them first. The estimation of PI can be done in 2 steps and since it is easier to get illumination-invariant descriptors, the first step should be pose estimation [3–5]. In this paper, we aim at the estimation of illumination conditions under certain poses. That is, if the pose of an input image is known, our method can estimate the illumination directions of that pose.

There are many state-of-the-art works related to illumination estimation for face recognition which can be roughly categorized into two categories, namely, the traditional 2D based methods [6–10] and the current popular 3D reconstruction based method [11–17]. As is indicated in [11], when PI conditions vary, the intensity of 2D face image changes greatly, so 2D appearance based techniques cannot work. Though some 2D methods based on multiview [6] can handle PI changes to some extent, we can say that only by relying on 3D information to ameliorate 2D image appearance we can solve this problem in nature.

However, there are 3 main problems with 3D reconstruction based method: (1) generalization problem—all 3D reconstruction based methods require that the subject to be recognized be also in the training set, which is suitable for face recognition. But this requirement cannot be met in subject-independent expression recognition. (2) The 3D reconstruction process (e.g., 3D morphable model) itself is computationally expensive [13]; (3) in order to estimate the illumination of an input image, we need to match the input image with all the lighting images of *M* training samples' 3D reconstructed face models. Suppose that there are totally *N* kinds of illumination conditions; then, the computational complexity will be *O*(*M* × *N*).

In this paper, we propose a subject-independent illumination estimation method, which can solve the generalization problem by RF and clustering technique with a complexity of *O*(*C* × *N*), where *C*, as a constant, is the number of clusters. First, all 256 training 3D face models are adaptively categorized into a certain amount of facial structure types by *k*-means clustering, so people with the similar facial appearance are clustered together; then the RF of each cluster is generated to represent the typical facial appearance of that cluster. By rotating all RFs to a certain pose, illuminating them with a series of illumination conditions, and projecting them to 2D, we get all the lighting face images as our training set. Finally, we select the most discriminative saltire-over-cross features to train a group of SVM classifiers and get satisfactory estimation accuracy when estimating a number of test sets including images generated from 64 3D face models kept for testing, CAS-PEAL face database, and CMU PIE database, as well as a small test set created by ourselves.

**Figure 1:**
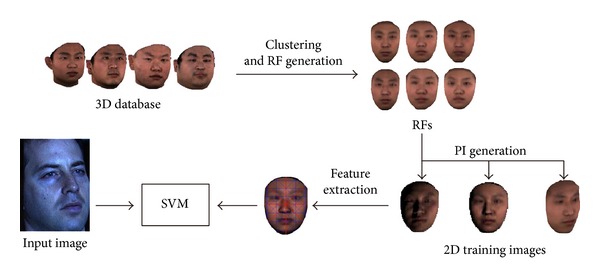
System overview.

Figure 1 gives an overview of our RF and clustering based system.

The rest of the paper is organized as follows. In the next section, we give a brief introduction of the dataset we utilized and the preprocessing method. In Section 3, we apply adaptive *k*-means clustering to solve the generalization problem.  Section 4 presents the concept of 3D representative face, namely, RF. In Section 5, we introduce the saltire-over-cross feature and the SVM classifier we used in illumination estimation. In Section 6, we show the experimental results on several face databases and proposed a two-stage classification framework to promote the recognition accuracy on CAS-PEAL database. Conclusions are given in Section 7.

## 2. Dataset and Preprocessing

### 2.1. BJUT-3D Face Database

The dataset used to generate RFs in our experiments is BJUT-3D Face Database [18], in which each 3D face model consists of 50000–80000 vertices and more than one hundred thousand triangular patches. Each vertex has its texture information, described in RGB format, as formulated below, for the *i*th face model. Consider the following.

Shape vector consists of *n*_*i*_ vertices:

(1) $$\begin{array}{c} S_{i}={\left(X_{i1},Y_{i1},Z_{i1},\ldots ,X_{in_{i}},Y_{in_{i}},Z_{in_{i}}\right)}^{T}. \end{array}$$

Texture vector represents the color of the *n*_*i*_ vertices:

(2) $$\begin{array}{c} T_{i}={\left(R_{i1},G_{i1},B_{i1},\ldots ,R_{in_{i}},G_{in_{i}},B_{in_{i}}\right)}^{T}. \end{array}$$

Triangular patch vector represents the *m*_*i*_ triangular patches:

(3) $$\begin{array}{c} {\text{TP}}_{i}=\left(V_{{il}_{1}},V_{{il}_{2}},V_{{il}_{3}},\ldots ,V_{{im}_{i1}},V_{{im}_{i2}},V_{{im}_{i3}}\right). \end{array}$$

**Figure 2:**
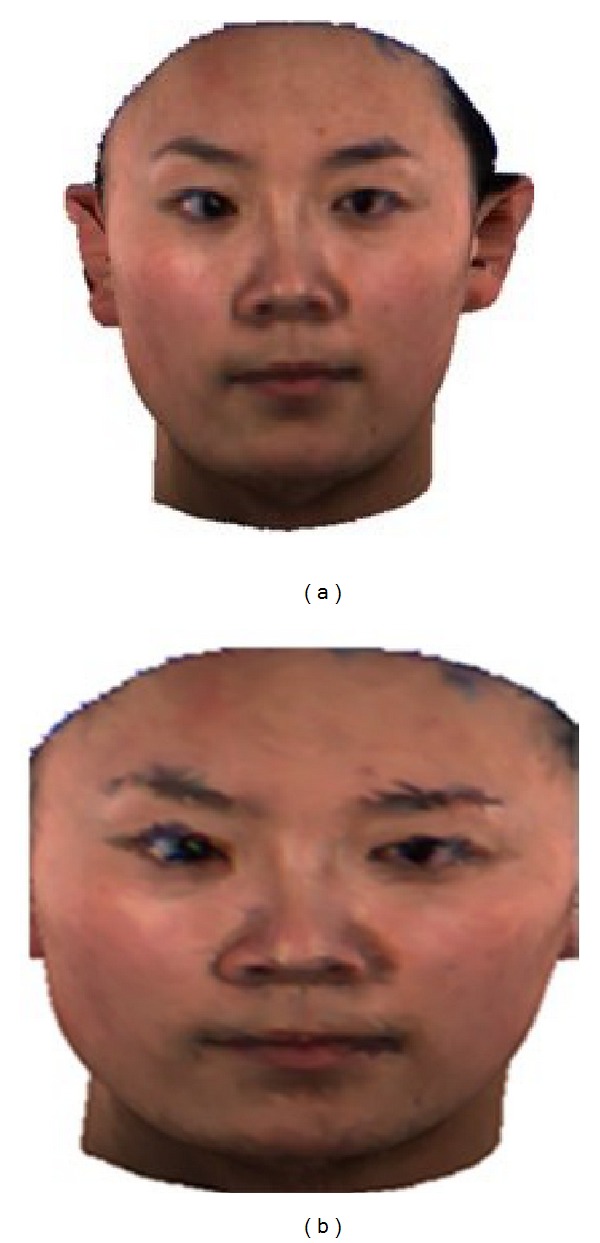
3D face model (a) before mesh simplification and (b) after mesh simplification.

We randomly select 400 subjects, 199 males and 201 females in the beginning, but, due to the inner problem of some models (burr and triangular patch relation error), we just keep 320 good models in the end as the dataset for our experiments; Figure 2(a) shows an example face model.

The training set should contain as many types of facial appearance as possible in order to make our RF and clustering based method work well. So a relative large number of 3D face models are required to generate the training set. All the 320 3D face models are divided into 2 parts randomly; 256 models are used to generate the representative faces for training. The other 64 models are kept to generate test images with variant illuminations for testing the generalization ability of our method.

### 2.2. Mesh Simplification and Pixel-Wise Correspondence

As described in Section 2.1, the original 3D face model has very high precision, which is unnecessary and also brings huge difficulty in computing. Many related researches have shown that precision of 7000–10000 vertices is appropriate [11, 19] for automatic face analysis. So, in this paper, all models are simplified to 8000 vertices (as shown in Figure 2(b)) using Garland's mesh simplification algorithm [20] to make a trade-off between computational complexity and model precision.

Another useful step to make 3D face vectors computable is pixel-wise correspondence, which is necessary for generating RF in Section 4. In this paper, resampling based method is used for pixel-wise correspondence [21, 22].

After mesh simplification and pixel-wise correspondence, the shape and texture information of the *i*th 3D face model can be formulated using two 3 × 8000-dimension vectors, *S*_*i*_ and *T*_*i*_:

(4) $$\begin{array}{c} S_{i}={\left(X_{i1},Y_{i1},Z_{i1},\ldots ,X_{in},Y_{in},Z_{in}\right)}^{T},\\ T_{i}={\left(R_{i1},G_{i1},B_{i1},\ldots ,R_{in},G_{in},B_{in}\right)}^{T}, \end{array}$$

where *n* = 8000.

The triangular patches can be computed using the Delaunay triangulation algorithm [23, 24] according to the face model's vertices information.

## 3. Adaptive 3D Face Clustering

It is the subject's 3D facial structure that determines the appearance (intensity distribution) of his/her photo under various illuminations, and facial organs such as eyes, nose, and mouth are the main cause of 3D structure difference among different people. Though people's facial appearances differ in thousands of ways, their facial structures can be classified into some main types according to the positions and shapes of their facial organs. By clustering all 256 3D face models according to the coordinates of their main facial organs, we actually cluster them into a number of facial structure types; namely, faces in one cluster are alike and each cluster represents a facial structure type (as shown in Figure 5, RFs of 6 clusters represent 6 different facial structure types).

### 3.1. Normalization

**Figure 3:**
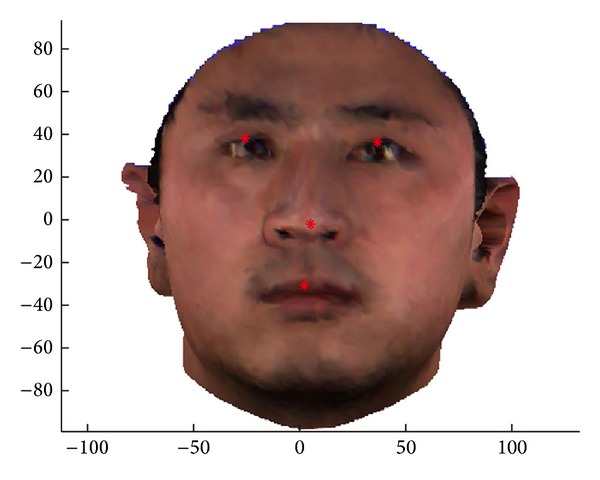
Four fiducial points.

Before clustering, we must obtain the coordinates of 4 facial fiducial points—two eyes, nose tip, and upper-lip tip—whose detection in a 3D face model is much easier than in a 2D image. The point with the greatest *z* value is nose tip, below which the first *z* peak value indicates the position of upper-lip tip. By projecting frontal 3D model to 2D image, we can detect the *x*-*y* coordinates of two eyes using gray-level projection method [25, 26] and get their *z* values back in the 3D model.  Figure 3 gives an illustration of 4 fiducial points detected in a face model.

Whether two subjects are alike has nothing to do with their head size and pose in an image. So our clustering algorithm should be scale and rotation invariant. Each model in the database undergoes a 3D transformation with a vertical stretch to map its 4 fiducial points to the same destination set of fiducial points. Mathematically, the four 3D fiducial points $\left(\vec{n_{1}},\vec{n_{2}},\vec{n_{3}},\vec{n_{4}}\right)$, for each model, are mapped to a destination set of 3D fiducial points $\left(\vec{m_{1}},\vec{m_{2}},\vec{m_{3}},\vec{m_{4}}\right)$. This mapping is given in

(5) {mixmiymiz}=T{nixniyniz1}, i=1,2,3,4,

where matrix *T* is defined as follows:

(6) $$\begin{array}{c} T=\left\{\begin{array}{cccc} s\left(\cos\theta_{y}\cos\theta_{z}\right) & -s\left(\cos\theta_{y}\sin\theta_{z}\right) & s\left(\sin\theta_{y}\right) & t_{x}\\ s\left(\cos\theta_{z}\sin\theta_{x}\sin\theta_{y}+\cos\theta_{x}\sin\theta_{z}\right) & s\left(\cos\theta_{x}\cos\theta_{z}-\sin\theta_{x}\sin\theta_{y}\sin\theta_{z}\right) & -s\left(\cos\theta_{y}\sin\theta_{x}\right) & t_{y}\\ s\left(\sin\theta_{x}\sin\theta_{z}-\cos\theta_{x}\cos\theta_{z}\sin\theta_{y}\right) & s\left(\cos\theta_{z}\sin\theta_{x}+\cos\theta_{x}\sin\theta_{y}\sin\theta_{z}\right) & s\left(\cos\theta_{x}\cos\theta_{y}\right) & t_{z} \end{array}\right\}. \end{array}$$

Here, ${\vec{m}}_{i}$ is the mean of ${\vec{n}}_{i}$ for all the *M* face models:

(7) $$\begin{array}{c} \vec{m_{i}}=\frac{1}{M}\overset{M}{\underset{j=1}{\sum }}{\text{Face}}_{j}\cdot \vec{n_{i}},\;i=1,2,3,4. \end{array}$$

Using 256 3D models, the best transformation matrix is found by optimizing the 7 parameters (*t*_*x*_, *t*_*y*_, *t*_*z*_, *θ*_*x*_, *θ*_*y*_, *θ*_*z*_, *s*) to minimize the fitting error, *E*_fit_, as defined below. There are 3 translation parameters (*t*_*x*_, *t*_*y*_, *t*_*z*_), 3 rotation parameters (*θ*_*x*_, *θ*_*y*_, *θ*_*z*_), and one stretch parameter *s*:

(8) $$\begin{array}{c} E_{\text{fit}}=\overset{4}{\underset{i=1}{\sum }}\sqrt{{\left(n_{ix}-m_{ix}\right)}^{2}+{\left(n_{iy}-m_{iy}\right)}^{2}+{\left(n_{iz}-m_{iz}\right)}^{2}}. \end{array}$$

Then, we have all models' nose tips aligned to a base point (*x*_0_, *y*_0_, *z*_0_). Now, the coordinates of the remaining three fiducial points are used as features for a facial structure. They are then arranged into a vector $\vec{v}$:

(9) $$\begin{array}{c} \vec{v}={\left(\begin{array}{ccccccccc} x_{1} & y_{1} & z_{1} & x_{2} & y_{2} & z_{2} & x_{3} & y_{3} & z_{3} \end{array}\right)}^{9}. \end{array}$$

### 3.2. Adaptive *k*-Means Clustering

**Figure 4:**
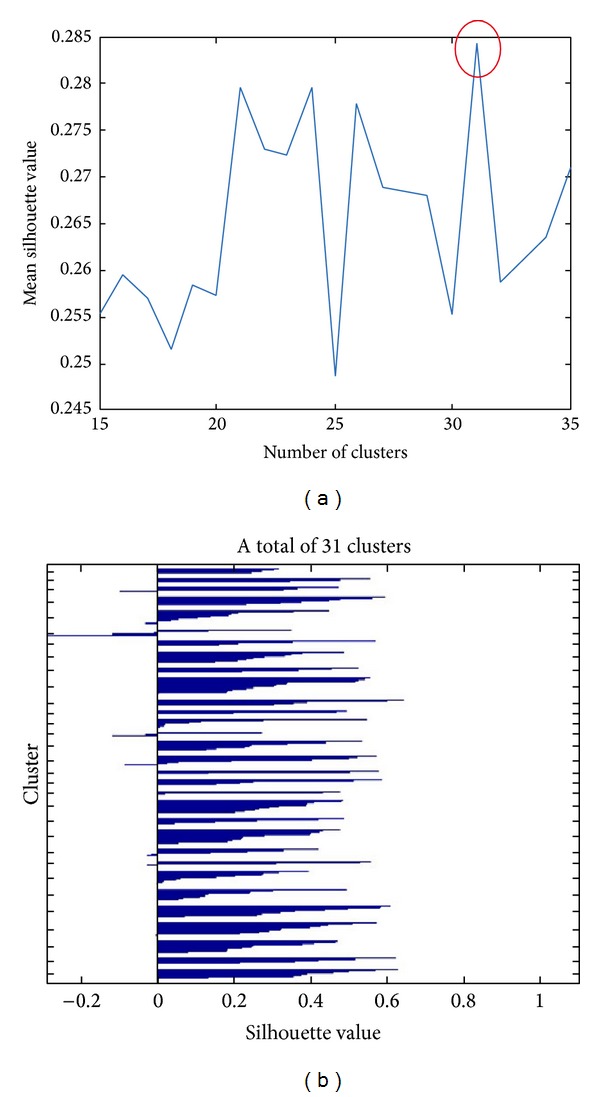
(a) Choice of clusters numbers and (b) silhouette value for 31 clusters.

It is difficult to decide an appropriate cluster number for *k*-means clustering algorithm without a good understanding of the inner structure of the data. Usually a better choice of cluster number is crucial to the clustering result. In this paper, we adaptively get the cluster number between 15 and 35 following the maximum mean silhouette value principle. During clustering, we repeat 5 times with different starting points in the case of local minima. Finally, we get the cluster number 31 as shown in Figure 4(a).

To have an idea of how well separated the resulting clusters are, see Figure 4(b) for a silhouette plot. The silhouette plot displays a measure of how close each point in one cluster is to points in the neighboring clusters. This measure ranges from +1, indicating points that are very distant from neighboring clusters, through 0, indicating points that are not distinctly in one cluster or another, to −1, indicating points that are probably assigned to the wrong cluster.

## 4. Generating RF

Now, we have 31 clusters. Faces within each cluster are alike and each cluster represents a facial structure type. The next step is to find a representative face that can represent its cluster's facial structure type.

A 3D average face represents a kind of 3D stable structure hidden behind all individual faces that contribute in computing it. We believe that, for the need of illumination estimation, it is stable and representative enough to approximate all individual faces belonging to its facial structure type. So we generate an average face for each of the 31 clusters to represent 31 types of facial structures.

The average face can be computed as follows:

(10) $$\begin{array}{c} \text{avgFace}\cdot \text{Shape}=\frac{\left(\sum_{i=1}^{M}S_{i}\right)}{M},\\ \text{avgFace}\cdot \text{Texture}=\frac{\left(\sum_{i=1}^{M}T_{i}\right)}{M}, \end{array}$$

where *S*_*i*_ and *T*_*i*_ are the shape and texture vectors of the *i*th 3D face model, respectively.

The triangular patches can be computed using the Delaunay triangulation algorithm [23, 24] according to the average face's vertices information.

**Figure 5:**
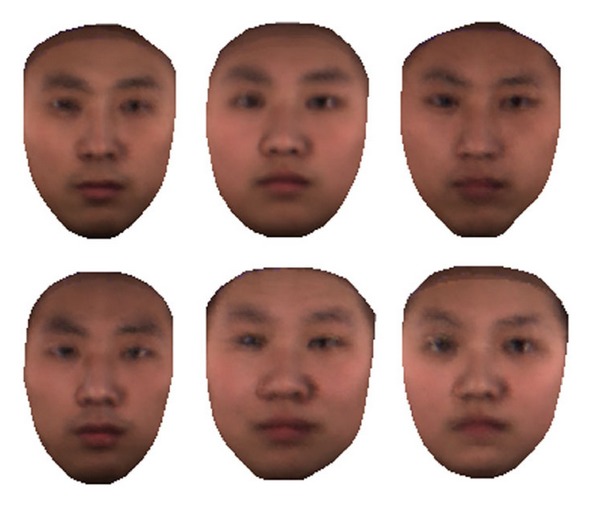
Six RFs of six clusters representing six different facial structure types.

We refer to [27] to generate the average 3D face. For each cluster, we generate an average 3D face, namely, RF, to represent the facial structure type of that cluster. For illustration, Figure 5 shows 6 RFs out of a total of 31.

## 5. Feature Selection and Classification

### 5.1. Generating Training Set

The training set is generated from the 31 RFs by rotating them to certain poses and illuminating them with certain lights. For instance, 13 kinds of illuminations are defined in the experiments of Section 6.1 as shown in Figure 8. First, we rotate all 31 RFs to a certain pose and illuminate each of them with the 13 kinds of illuminations defined in Figure 8. Then, we map all 31 × 13 illuminated RFs into 2D to get the training images for our 13-class problem. Each class has 31 training sample images. For a new test sample, we can always expect that there is a facial structure type it belongs to in our 31 RFs, and so each class has a training sample from that facial structure type, from which we get the generalization ability. This is the essential of our RF and clustering based method.

**Figure 6:**
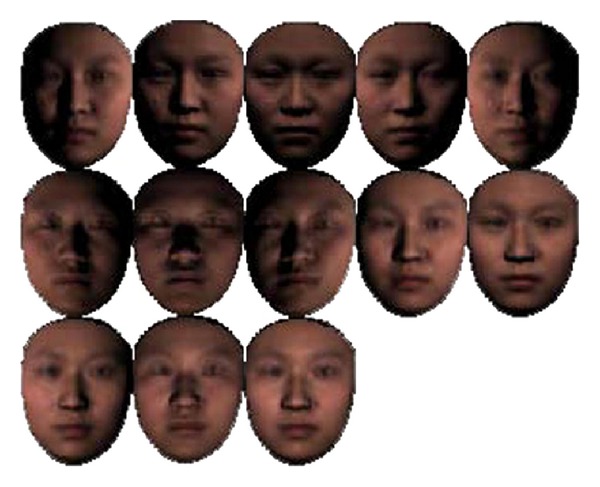
A representative face under 13 illuminations.

Figure 6 gives an illustration of some training images generated from one RF under 13 illuminations with frontal view.

### 5.2. Saltire-over-Cross Feature

In a pattern recognition problem, it is of great significance to get the most discriminative feature for classification. In this paper, we select pixels which are most sensitive to illumination changes as feature. We call it “saltire-over-cross feature” because its shape is like the symbol on a Union Jack.

**Figure 7:**
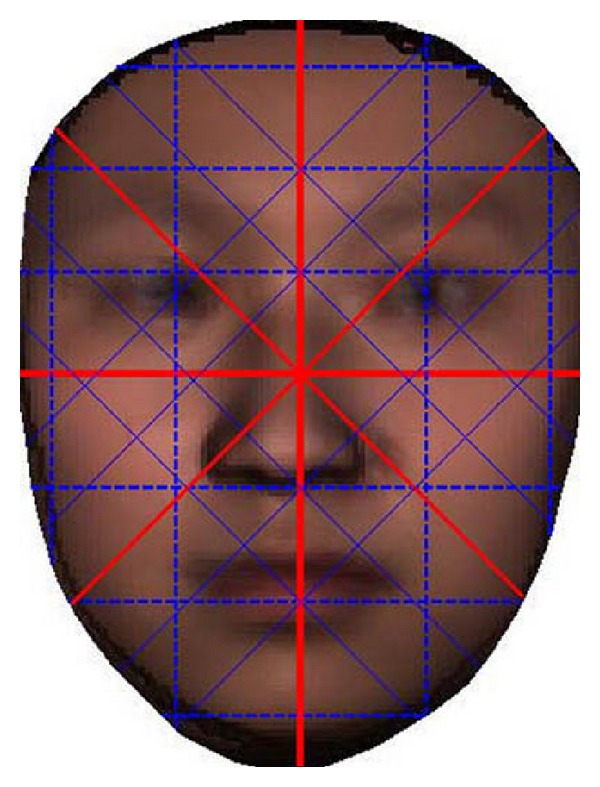
Saltire-over-cross feature—the gray values of pixels in the 22 pixel lines are selected as features.

As illustrated in Figure 7, four continuous lines form the saltire-over-cross symbol. We select 22 pixel lines (4 continuous lines plus 18 dashed lines) of 4 directions from 2D training images:

1. horizontal (7 pixel lines),
2. vertical (5 pixel lines),
3. +45 degree diagonal (5 pixel lines),
−45 degree diagonal (5 pixel lines).

Using general face detection algorithms to locate face region in an image, get the centroid of the region as the middle of the “saltire-over-cross” (continuous line in Figure 7). Split its upper, nether, left, and right region into several parts equally as illustrated in Figure 7. Resample corresponding pixel lines of different faces to make them the same dimension. At last, we arrange these 22 pixel lines to form one feature vector.

We also try using all pixels within the facial region and the concatenated histograms of image partitions [28, 29] as features. As shown in Table 1, compared with the regional feature and partitioned histogram feature, our saltire-over-cross feature can get better recognition accuracy when estimating the generated test set with frontal view in spite of its much lower dimension.

### 5.3. Support Vector Machines

Unlike many traditional classifiers that aim at minimizing the Empirical Risk, SVM [30, 31] approaches the classification problem as an approximate implementation of the structural risk minimization (SRM) induction principle [32, 33], which may mean better generalization ability.

In this paper, C-SVM with the radial basis function (RBF) kernel is used as our classifier. There are two parameters with C-SVM with RBF kernels, *C* and *γ*, where *C* > 0 is the penalty parameter of the error term and *γ* is the parameter for the RBF kernel; consider the following:

(11) $$\begin{array}{c} K\left(x_{i},x_{j}\right)=\exp\left(-\gamma {||x_{i}-x_{j}||}^{2}\right),\;\gamma >0. \end{array}$$

Fivefold cross-validation and the grid-search technique described in [34] are used here to find the best *C* and *γ* for our problem and it is when *C* = 8 and *γ* = 0.0001220703125 that the best estimation accuracy can be achieved.

### 5.4. Multiclass Classification

SVM, as explained above, is suitable only for binary classification, while our illumination estimation is an *N*-class problem, where *M* is the number of illuminations. However, there are many techniques that can extend SVM to handle a multiclass problem. In our experiment, we have tried three techniques including (1) “one-against-one” voting strategy [35]; (2) “one-against-one” eliminating strategy [36]; and (3) error-correcting output codes (ECOC) [37].

In a voting strategy, each binary classification is considered to be a vote where votes can be cast for all data points—at the end, a point is designated to be in a class with maximum number of votes, while, in an eliminating strategy, the margin size of each dichotomy is regarded as the classification confidence of that dichotomy. All dichotomies are sorted by their confidence, and, in each binary classification, one class is eliminated (see the paper in [36] for a detailed description).

When we use the “one-against-one" approach [36] in which *N*(*N* − 1)/2 classifiers are constructed and each one trains data from two different classes, satisfactory results can be obtained with both voting strategy and eliminating strategy with the performance of eliminating strategy being a little higher than the one of the voting strategy (96.88% versus 96.75% when estimating illuminations with frontal view in the experiments of Section 6.1). In our opinion, this may be due to the reason that with voting strategy in each binary classification the only information we can get is yes or no (+1 or −1), while with the eliminating strategy a real value between −1 and +1 (yes if > 0, no if < 0) is given as the confidence of that classification. Though this brings no difference in a 2-class problem, more information is provided for a multiclassification. However, the expected results are not obtained using ECOC.

## 6. Experimental Evaluation and Analysis

To test the validity of our illumination estimation method, several experiments are conducted on different datasets, such as images generated from the 64 3D face models kept for testing, CAS-PEAL face database, and CMU PIE database, as well as a small test set created by ourselves.

### 6.1. Illumination Estimation on Generated Test Set

**Figure 8:**
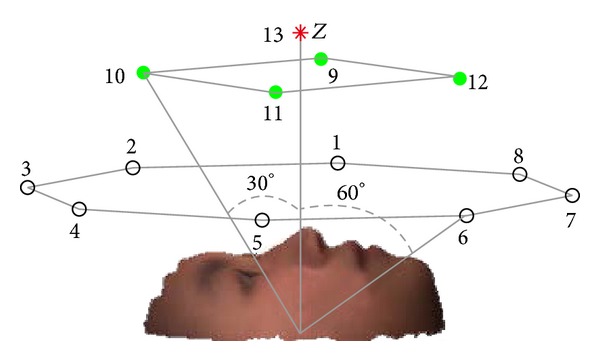
Positions of the 13 lamp-houses.

**Figure 9:**
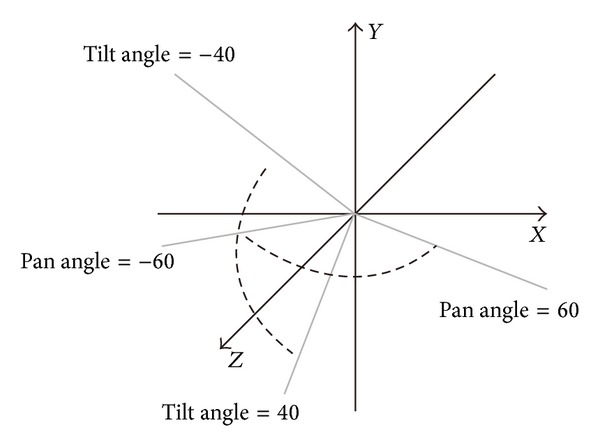
Tilt angle and pan angle in pose definition.

In this experiment, we define 13 lamp-house positions each for an illumination class. As shown in Figure 8, the farther the lamp-house is, the less impact it has on image intensity; the number of lamp-houses decreases as distance increases. All lighting images under a series of poses with pan angle spanning from −60° to 60° and tilt angle spanning from −40° to 40° are tested. See Figure 9 for illustration.

For each test, two estimation results are given; one is for the 3D face models participating in the generation of RFs, which are projected with 13 illuminations into 2D to form 256 × 13 images, called group-I, and the other is for the 64 × 13 test images, called group-II. Some typical results are outlined in Table 2. Data are formatted as group-I/group-II. We omit the results when pan angle is −30° or −60° because of symmetry.

From the estimation results shown in Table 2, it can be observed that both accuracies of group-I and group-II are satisfactory. When estimating samples in group-I, the accuracy is a little higher, which supports our first argumentation—it is a person's 3D facial structure that determines the appearance (intensity distribution) of his/her photo under variant illuminations and RF can represent the 3D facial structure of all 3D face models contributing to computing it perfectly.

Though test images in group-II have nothing to do with the generation of RF, we achieve comparable results when estimating samples in group-II. Actually, the accuracy of group-II is only a little bit lower than that of group-I in the large (sometimes even a little bit higher), which supports our second argumentation—there are some main types of facial structures, and the clustering technique does provide our illumination estimation system with a good generalization ability.

### 6.2. Illumination Estimation on CAS-PEAL Face Database

In the experiments of Section 6.1, the test set consisted of 2D lighting images generated from 3D models, in which the imaging conditions are the same as the training set, while these generated images are different from those illuminated photos taken from real scene.

To further enhance the robustness of our system, we conduct experiments on CAS-PEAL face database from the Chinese Academy of Sciences [38]. The large-scale CAS-PEAL face database consists of 99450 facial images of 1040 Chinese individuals with four principal variations of pose, expression, accessory, lighting, and so forth. In this experiment, we select 150 facial images of more than 10 people, each with 15 or less lighting conditions (some subjects have less than 15 lighting images).

**Figure 10:**
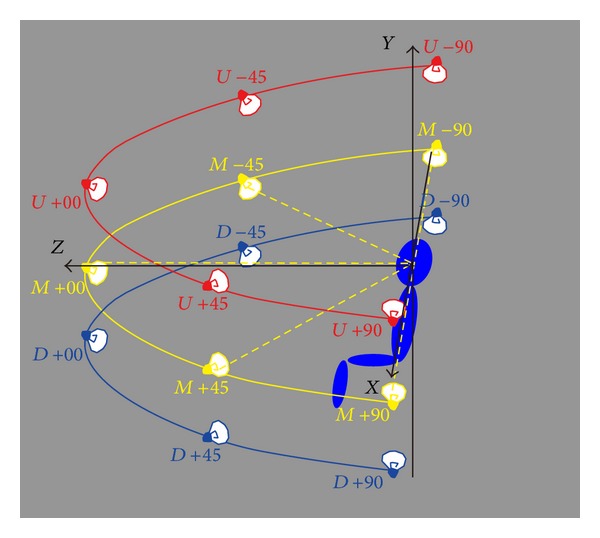
Lamp-house positions of CAS-PEAL face database.

Since the illumination positions of images in CAS-PEAL face database are different from those we set in Section 6.1, we rearrange 15 lamp-house positions located in three planes (*U*, *M*, and *D*) to be consistent with the CAS-PEAL test set, as illustrated in Figure 10.

**Figure 11:**
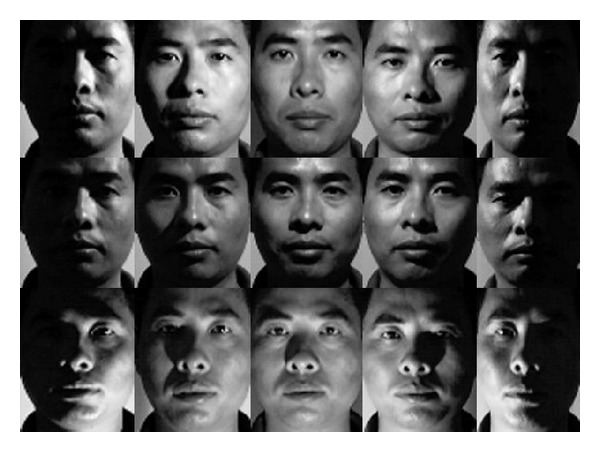
Normalized facial images from CAS-PEAL face database.

To be consistent with the sample images from training set, we interactively select the face regions and normalize all face images to uniform size as shown in Figure 11.

Finally, a 57.33% recognition accuracy is achieved when estimating 15 illumination conditions with our 3D representative face and clustering based method. To analyze experimental results, we print the 15 × 15 error matrix ErrMat as shown in Table 5, in which ErrMat(*i*, *j*) stands for the number of misclassified samples from class-*i* to class-*j*. Consider the following.

As can be seen, elements in the right part of ErrMat (column index > 10) have higher values, which indicates that some lamp-houses of the *U* and *M* planes are misclassified to lamp-houses of *D* plane. Furthermore, the misclassified class labels have the same *X*-*Z* coordinates with the correct labels, only different in *Y* values. The main reason is that there are significant differences between the test images from CAS-PEAL database and the training set generated from 3D face models, as summarized below.

1. All individuals in training set have their hair covered during the image acquisition, so no hair is shown in the training images, while most subjects in the images of test set have their forehead masked by hair, which results in low intensity in the upper area of image, and it seems like the lamp-house is in the nether *D* plane.
2. No accouterments can be found in training images, while some individuals in test set are wearing some accouterments, such as glasses.

### 6.3. Improvements with Two-Stage Classification

A two-stage recognition framework is presented to further promote the recognition performance on the test set from CAS-PEAL database in this paper. In the first stage, we use saltire-over-cross features discussed in Section 5.2 to distinguish horizontal positions of lamp-houses. In the second stage, we use the 5 vertical pixel lines of saltire-over-cross feature to further classify vertical positions of lamp-houses. The final decision *C* is formulated as follows:

(12) $$\begin{array}{c} C\;=\;\left(C_{1}\mathrm{mod}m\right)+\;\left(C_{2}-1\right)*m, \end{array}$$

where *C*_1_ is the output class label of the first stage, *C*_2_ is the computed class label of the second stage, and *m* is the number of horizontal lamp-house positions per plane. In our experiments, in Section 6.2, *m* equals 5.

Since the total number of classes is reduced greatly (only three vertical lamp-house positions) in the second stage, the classifier has more margin to distinguish one class from another. In this way, the vertical angle of the lamp-house positions can be estimated more accurately.

**Figure 12:**
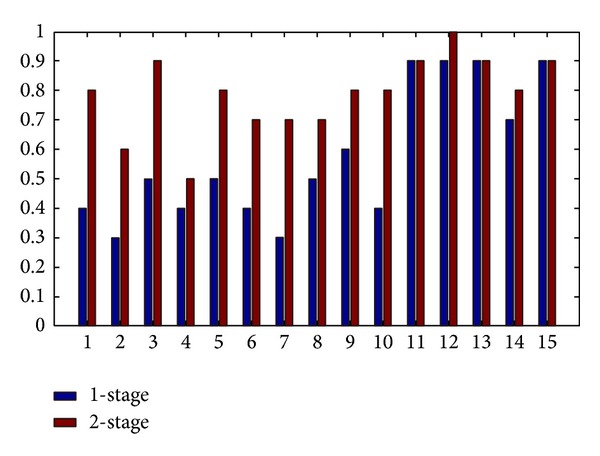
Accuracy comparison for each illumination class.

It can be observed in this experiment that the performance on CAS-PEAL database can be promoted greatly with two-stage classification. The overall accuracy can be promoted from 57.33% to 78.67%. As illustrated in Figure 12, recognition accuracy for each illumination class is compared with the results in Section 6.2.

### 6.4. Illumination Estimation on Small Image Set with Expressions

**Figure 13:**
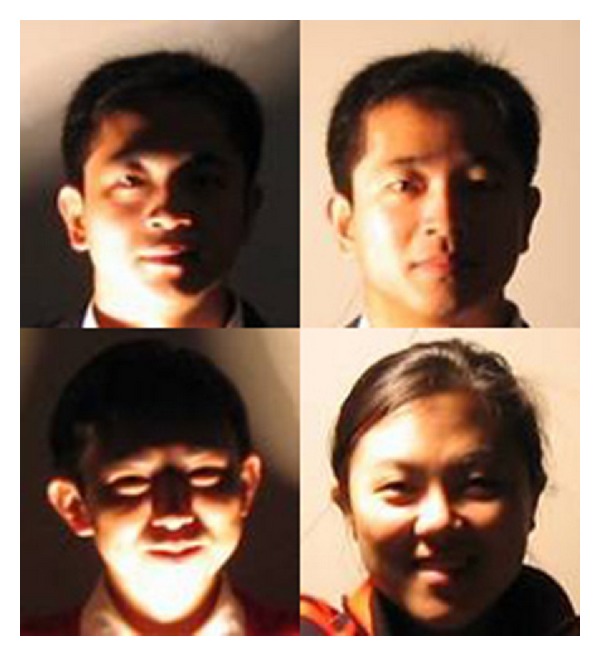
Example images from the small dataset collected by ourselves.

Neither CAS-PEAL nor CMU PIE face database provides images with both illumination and expression variations at the same time. Therefore, we create a small set of images by ourselves in this experiment, which contains more than 40 images of 5 people posing several expressions under some illuminations defined in Figure 10; see Figure 13 for illustration.

In experiments, 72% recognition accuracy is achieved on the small image set by our two-stage illumination estimation framework based on 3D RF and clustering.

### 6.5. Comparing with Related Works

In [17], Huang et al. proposed a 3D reconstruction based method, which gives an estimation of illumination directions using angles rather than class labels. In order to compare with their work, we also define 2 test sets: (1) face images with certain pose and illumination generated from the 64 3D face models kept for testing with the pose changing from −90° to +90° horizontally and from −45° to +45° vertically and the illumination direction changing from −45° to +45° horizontally and from −45° to +45° vertically and (2) a portion of CMU PIE database [39] which contains face images captured from 3 cameras (camera index: 34, 27, and 05) under 3 illumination directions (flash index: 09, 11, and 21).

We set head pose and lighting directions the same as in Huang et al. experiments [17] in both training and testing but only estimate illuminations; Table 3 shows the estimation results on test set (1) of our method versus Huang et al., formatted as ours/Huang et al., given by mean value and standard deviation of estimation error in angles.

It turns out that our method achieves comparable performance when estimating horizontal illumination changes without 3D reconstruction. Meanwhile it outperforms Huang et al. method [17] prominently when estimating vertical changes.

**Figure 14:**
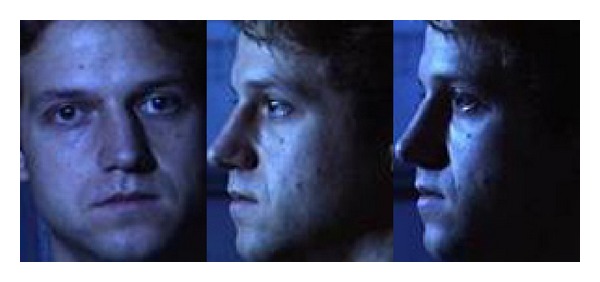
Example cropped images from the CMU PIE face database.

In Table 4, we show the estimation results on test set (2) of our method versus Huang et al.'s, formatted as ours/Huang et al.'s. The original face images are cropped and normalized to uniform size to be consistent with the sample images from the training set, as shown in Figure 14.

As shown in Table 4, the RF and clustering based approach presented in this paper achieve comparable results with Huang et al.'s method, while saving the trouble of 3D reconstruction and reducing classification complexity from *O*(*M* × *N*) to *O*(*C* × *N*).

## 7. Conclusions and Future Works

In this paper, a subject-independent illumination estimation method is proposed, which can solve the generalization problem by using RF and clustering technique with a complexity of *O*(*C* × *N*). Satisfactory performance has been achieved when we conduct experiments on several datasets, including images generated from 64 3D face models kept for testing, CAS-PEAL face database, and CMU PIE database, as well as a small test set created by ourselves. A two-stage classification framework is introduced when estimating illuminations of real scene images from CAS-PEAL database.

When estimating test images with expression variations (e.g., Section 6.4), the performance is inferior to those without expression variations (e.g., Section 6.3). This is due to the fact that our training images generated by 3D face models are all with neutral expression. So our future work will be to make sure that there is a training image with the same expression as the input testing image, and this can be done by performing expression synthesis [40–42] during the generation of the training set.

The illumination estimation algorithm presented in this paper can be applied to a broad range of applications in face recognition and expression recognition to estimate the illumination conditions as long as the objects to be classified are at similar location, orientation, and scale in both the training and the test images. So, in experiments, we need to manually crop and normalize the test images. However, if the system is used in conjunction with appropriate segmentation and rectification algorithms, then these constraints can be removed.

## Figures and Tables

**Table 1:** Comparison of different features.

Feature	Dimension	Accuracy	Description
Saltire-over-cross feature	2825	96.27	The downsampled saltire-over-cross feature.
Saltire-over-cross feature	5649	96.94	The original saltire-over-cross feature.
Regional feature	2856	94.47	Select pixels of the whole facial region as feature, and resample the feature vector to 2856-dimension.
Regional feature	5820	94.95	Select pixels of the whole facial region as feature, and resample the feature vector to 5820-dimension.
Partitioned histogram	11520	89.78	Partition the whole facial region into some blocks, extract histogram from each block, and concatenate all histograms to form a feature vector.

**Table 2:** Illumination estimation performance under certain poses (1st row indicates pan angle; 1st column indicates tilt angle).

Angle	0°	30°	60°
−40°	92.22%/91.59%	93.60%/94.23%	92.01%/90.38%
−20°	94.35%/92.67%	92.07%/93.60%	93.90%/92.07%
0°	97.39%/96.88%	96.94%/96.51%	95.10%/93.99%
20°	90.53%/89.54%	90.99%/90.87%	88.43%/88.46%
40°	82.48%/82.57%	80.38%/81.01%	73.23%/72.84%

**Table 3:** Average estimation error with varying illumination under a series of poses. ±90°, I-2-H, indicates horizontal (H) (V for vertical) changes from −90° to 90° with 2° increments (I).

Pose range	Illumination range	Average error	Standard deviation
±90°, I-10-H	±45°, I-4-H	6.41°/7.32°	6.32°/6.36°
±90°, I-20-H	±45°, I-10-H	9.74°/8.27°	13.53°/7.19°
±45°, I-10-V	±45°, I-10-V	2.03°/9.12°	3.07°/10.40°

**Table 4:** Estimation accuracy by computing the relative horizontal angles for different flashes. Camera numbers are 5, 27, and 34. Illumination numbers are 9, 11, and 21. The last column (computed values) is calculated from geometric information provided in the database.

	Average estimation	Standard deviation	Computed values
Illumination: 11⇔9	8.46°/10.28°	34.52°/20.04°	16.53°
Illumination: 9⇔21	2.12°/1.89°	7.28°/5.63°	4.30°

**Table 5:** The error matrix.

$\text{ErrMat}=\begin{array}{ccccccccccccccc} 0 & 1 & 0 & 0 & 0 & 1 & 0 & 0 & 0 & 0 & 3 & 1 & 0 & 0 & 0\\ 1 & 0 & 0 & 0 & 0 & 0 & 0 & 0 & 0 & 0 & 1 & 4 & 1 & 0 & 0\\ 0 & 0 & 0 & 0 & 0 & 0 & 0 & 0 & 0 & 0 & 0 & 1 & 4 & 0 & 0\\ 0 & 0 & 1 & 0 & 0 & 0 & 0 & 0 & 0 & 0 & 0 & 0 & 2 & 3 & 0\\ 0 & 0 & 0 & 0 & 0 & 0 & 0 & 0 & 0 & 0 & 0 & 0 & 0 & 1 & 4\\ 1 & 0 & 0 & 0 & 0 & 0 & 1 & 0 & 0 & 0 & 3 & 1 & 0 & 0 & 0\\ 0 & 0 & 0 & 0 & 0 & 1 & 0 & 1 & 0 & 0 & 1 & 4 & 0 & 0 & 0\\ 0 & 0 & 1 & 0 & 0 & 0 & 0 & 0 & 1 & 0 & 0 & 1 & 2 & 0 & 0\\ 0 & 0 & 0 & 0 & 0 & 0 & 0 & 1 & 0 & 0 & 0 & 0 & 0 & 2 & 1\\ 0 & 0 & 0 & 0 & 1 & 0 & 0 & 0 & 1 & 0 & 0 & 0 & 0 & 1 & 3\\ 0 & 0 & 0 & 0 & 0 & 0 & 0 & 0 & 0 & 0 & 0 & 1 & 0 & 0 & 0\\ 0 & 0 & 0 & 0 & 0 & 0 & 1 & 0 & 0 & 0 & 0 & 0 & 0 & 0 & 0\\ 0 & 0 & 0 & 0 & 0 & 0 & 0 & 0 & 0 & 0 & 0 & 1 & 0 & 0 & 0\\ 0 & 0 & 0 & 0 & 0 & 0 & 0 & 0 & 1 & 0 & 0 & 0 & 1 & 0 & 1\\ 0 & 0 & 0 & 0 & 0 & 0 & 0 & 0 & 0 & 0 & 0 & 0 & 0 & 1 & 0 \end{array}$
